# An alternative approach to combat multidrug-resistant bacteria: new insights into traditional Chinese medicine monomers combined with antibiotics

**DOI:** 10.1007/s44307-025-00059-7

**Published:** 2025-02-07

**Authors:** Cunchun Dai, Ying Liu, Fan Lv, Ping Cheng, Shaoqi Qu

**Affiliations:** 1 Animal-Derived Food Safety Innovation Team, College of Animal Science and Technology, Anhui Agricultural University, Hefei, 230036 China; 2 College of Materials and Chemistry, Anhui Agricultural University, Hefei, 230036 China

**Keywords:** Multidrug-resistant bacteria, Traditional Chinese medicine monomers, Antibiotics, Combination therapy, Antibacterial mechanism

## Abstract

Antibiotic treatment is crucial for controlling bacterial infections, but it is greatly hindered by the global prevalence of multidrug-resistant (MDR) bacteria. Although traditional Chinese medicine (TCM) monomers have shown high efficacy against MDR infections, the inactivation of bacteria induced by TCM is often incomplete and leads to infection relapse. The synergistic combination of TCM and antibiotics emerges as a promising strategy to mitigate the limitations inherent in both treatment modalities when independently administered. This review begins with a succinct exploration of the molecular mechanisms such as the antibiotic resistance, which informs the antibiotic discovery efforts. We subsequently provide an overview of the therapeutic effects of TCM/antibiotic combinations that have been developed. Finally, the factors that affect the therapeutic outcomes of these combinations and their underlying molecular mechanisms are systematically summarized. This overview offers insights into alternative strategies to treat clinical infections associated with MDR bacteria and the development of novel TCM/antibiotic combination therapies, with the goal of guiding their appropriate usage and further development.

## Introduction

Antibiotics are among the most transformative discoveries of the twentieth century, revolutionizing the treatment of bacterial infections and profoundly impacting public health (Wang et al. 2022). Initially, antibiotics were instrumental in treating a spectrum of bacterial diseases, including pneumonia, tuberculosis, urinary tract infections, and skin infections. This breakthrough revolutionized medicine by providing effective therapies for the previously life-threatening conditions. Antibiotics quickly became the cornerstone of bacterial infection treatment, exert control over bacterial pathogenesis through mechanisms that disrupt cell integrity or inhibit essential growth and division processes (Muñoz et al. 2024; Steenbergen et al. 2005). The use of antibiotics has expanded to encompass various sectors, including human health care, animal breeding, agriculture, and industry (Smith et al. 2018). However, the widespread misuse and overuse of antibiotics have precipitated the emergence of resistance among microbial populations, so many drugs are ineffective against multidrug-resistant (MDR) bacteria (Pajares et al. 2022). In recent years, the alarming number of resistance mechanisms, such as New Delhi metallo-β-lactamase (NDM) and mobile colistin resistance (MCR), exacerbating the challenge of antibiotic resistance in pathogenic bacteria. This resistance poses significant hurdles in treating bacterial infections, even when antibiotics are used at clinically achievable concentrations.

The increasing challenge of antimicrobial resistance in public health calls for innovative regimens and approaches. Despite advancements in antimicrobial procedures (Simpkin et al. 2017), the Food and Drug Administration (FDA) has approved nearly 40 new antibiotics since 2000, and only a few of them have unique mechanisms of action (Rex et al. 2013). The high financial and time investments required for drug development are major factors that a coverage of contributing to the decline in new antibiotics, despite the evident medical need (Butler et al. 2020; Outterson et al. 2015). The antimicrobial development can take up to 10 years, and an estimated cost of US $800 million, which significantly reduces commercial benefits (Durand et al. 2019). Alternative therapies to antibiotics such as antigens, antibodies, probiotics, and vaccines, have been explored (Dickey et al. 2017; Johnson et al. 2017). Nevertheless, these alternative therapies are most effectively used as adjunctive or preventive measures in clinical settings, since their efficacy and safety as standalone treatments cannot be assured. Consequently, there is a pressing need to innovate new therapeutic approaches to combat resistant pathogens. In this context, combination therapy represents a promising treatment modality to circumvent the substantial investment for developing novel pharmaceuticals (Worthington et al. 2013).

Clinical practice and experimental studies have shown that many Chinese herbal medicines have antibacterial and bactericidal effects, such as *Coptis chinensis*, *Phellodendron chinensis*, *Skullcap*, *Forsythia*, and *Honeysuckle*, which have antibacterial effects on various bacteria but also have antibacterial effects on some antibiotic-resistant strains (Meng et al. 2024; Lee et al. 2017a, b). As a result, repurposing nonantibiotic drugs (such as traditional Chinese medicine (TCM) monomers) that have undergone rigorous toxicological and pharmacological analysis is an effective way to reduce the time, cost, and risk associated with traditional antibiotic innovation (Liu et al. 2019; Peyclit et al. 2019).

The integration of TCMs with antibiotics represents a novel strategy for combating MDR bacterial infections. TCMs not only offer advantages such as reduced side effects and minimized resistance development but also complement antibiotics in targeting resistant pathogens. This review delineates the molecular mechanisms underlying antimicrobial resistance in bacterial pathogens and explores diverse combination strategies involving antibiotics and TCM, elucidating their respective modes of action. Notably, the reintroduction of TCMs as innovative antibiotic adjuncts is emphasized, underscoring their advantages in advancing next-generation combination therapies. Furthermore, this review synthesizes the synergistic antibacterial mechanisms of TCMs against clinically significant drug-resistant pathogens, including both gram-negative and gram-positive bacteria. Finally, the review underscores the challenges associated with these combinatorial approaches and discusses future prospects for developing broad-spectrum combination therapies.

## Molecular mechanisms of antibiotic resistance

**Fig. 1:**
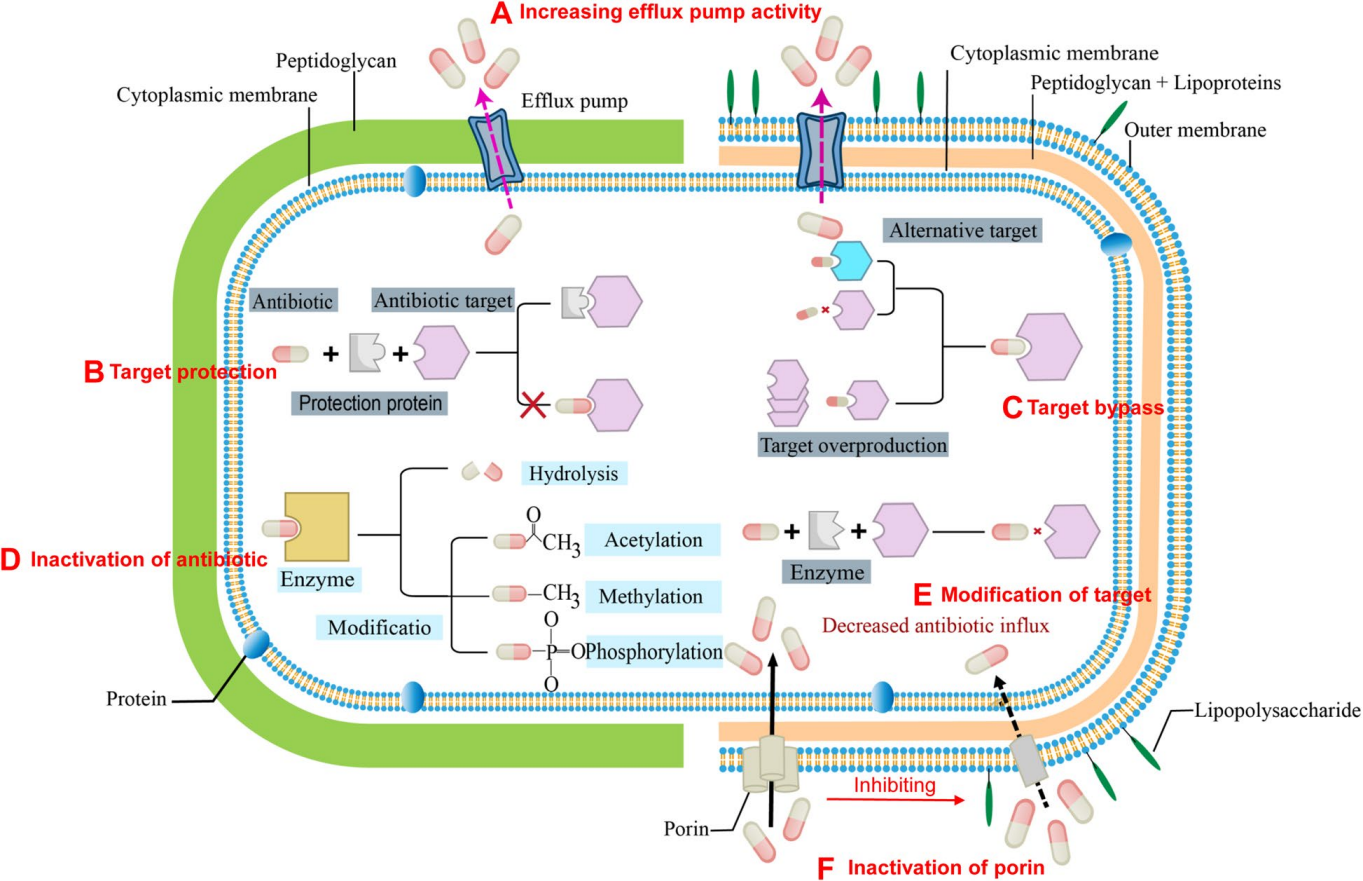
Molecular mechanisms of antibiotic resistance in Gram-positive and Gram-negative bacteria. **A** Decreasing the intracellular antibiotic accumulation through promoting the overexpression of efflux pumps; (**B**) Protecting the target of antibiotic through the target protection protein; (**C**) Overexpressing or replacing the target of antibiotic; (**D**) Hydrolyzing or modifying antibiotics by enzyme; (**E**) Modification of target site of antibiotic; (**F**) Preventing the entry of the antibiotic through reducing of the membrane permeability or downregulating/inactivating porin

To mitigate the impact of antibiotics, bacteria have evolved an array of strategies to increase their survival probabilities (Fig. 1). These strategies include both "intrinsic" mechanisms, where bacteria used inherent determinants of resistance to endure antibiotic exposure, and Max can "acquired" mechanisms, involving the acquisition of new genetic material conferring additional survival capabilities (Darby et al. 2023). Inherent resistance to certain antibiotics commonly occurs in specific bacterial species because of inherent structural or functional characteristics. For example, vancomycin (Jeffres et al. 2017) and rifampicin (Lee et al. 2017a, b) are highly effective against Gram-positive bacteria, but ineffective against Gram-negative bacteria. This is mainly due to the highly impermeable outer membrane (OM) of Gram-negative bacteria, which prevents the entry of antibiotics that are effective against Gram-positive bacteria (Konovalova et al. 2017). Biofilms are another form of intrinsic antibiotic resistance determinant (Hall et al. 2017). In addition to intrinsic resistance, bacteria can acquire foreign resistance genes through mechanisms such as horizontal gene transfer (such as coupling) (Husnik et al. 2018; Soucy et al. 2015) or chromosomal mutation (Arzanlou et al. 2017; Durão et al. 2018). In summary, antibiotic resistance can arise through three primary mechanisms: prevention of intracellular accumulation (via decreased permeability and antibiotic efflux), deactivation of antibiotics, and modification of the antibiotic target site (Darby et al. 2023). These adaptive strategies underscore the formidable challenges in combating bacterial infections, highlighting the imperative to understand the mechanisms of bacterial resistance to devise effective coping strategies.

### Prevention of intracellular antibiotic accumulation

The entry of antibiotics into cells to bind to their targets is crucial for their antibacterial activity (Richter et al. 2017). Gram-negative bacteria are characterized by a strong lipopolysaccharide outer layer that acts as a strong barrier against the entry of multiple molecules, especially antibiotics. Outer membrane porin (OMP) is a component of this lipopolysaccharide outer layer and plays a key role in regulating antibiotic influx into bacterial cells, a process that depends on the number and specific characteristics of these channels. In general, antibiotics with larger molecular sizes, greater negative charges, and significant hydrophobicity are less effective at crossing the outer membrane barrier (Ruggerone et al. 2013). Therefore, a reduction in or loss of porins leads to resistance to multiple antibiotics. For example, a decrease in porin production has been linked to the development of carbapenem resistance in *Enterobacterales* (Wozniak et al. 2012). A reduction in the number of OMPs in some bacteria, such as *Pseudomonas aeruginosa*, *Klebsiella pneumoniae*, and *Escherichia coli*, has been linked to antibiotic resistance (Nordmann et al. 2024; Belousov et al. 2023; Lumbreras-Iglesias et al. 2021). Specifically, the reduction in OMPs in *P. aeruginosa* has been shown to confer resistance to the antibiotic imipenem. Similarly, reductions in OMPs in *K. pneumoniae* have been associated with resistance to ceftazidime (Quinn et al.^1986^). In *E. coli*, reductions in OMPs have been implicated in resistance to quinolone antibiotics (Belousov et al. 2023). Additionally, some bacterial strains with mutations in OmpF and OmpC, the most abundant OMPs in gram-negative bacteria, have been found to be more resistant to beta-lactam antibiotics, such as penicillin, owing to the reduced permeability of the outer membrane (Hu et al. 2022). Collectively, these findings highlight the intricate relationship between OMP functionality and antibiotic susceptibility, underscoring the necessity of targeted research into porin-mediated pathways. Such investigations could inform the design of adjuvants or therapeutic strategies aimed at restoring or enhancing porin activity to counteract resistance.

Efflux pumps are integral membrane proteins that are mainly found in bacteria and are tasked with expelling toxins and harmful compounds from within the cell. These pumps play a key role in antibiotic resistance by reducing intracellular drug concentrations, thereby reducing therapeutic efficacy. Owing to their outer membrane, gram-negative bacteria exhibit increased antibiotic resistance, which is a strong barrier against many antibiotics. The efflux pumps the membrane-breaking antibiotic out of the cell by active injection to further exacerbate this resistance. These pumps, which are located primarily in the plasma membrane, use proton motive power to facilitate drug outflow, thereby enhancing bacterial survival against antibiotic challenges (Levy et al. 2002; Poole et al. 2002). The efflux pump family is divided into five families on the basis of its structure, transport substrate, and energy source: the major facilitator superfamily (MFS), ATP binding cassette (ABC), resistance nodulation cell division (RND), multidrug and toxic compound extrusion (MATE), and small multidrug resistance (SMR) families. The most well-studied RND pump is the RND pump, which is found only in gram-negative bacteria such as AcrAB-TolC in *E. coli* and MexAB-OprM in *P. aeruginosa* (Nishino et al. 2001; Poole et al.^1993^). RND pumps have large binding bags and can extrude a variety of antibiotics, making them the most important efflux pumps contributing to resistance to multiple drugs in pathogens (Yu et al. 2003). The multifaceted resistance conferred by these pumps poses a major challenge in clinical treatment, necessitating the development of efflux pump inhibitors to restore drug efficacy.

In gram-positive bacteria, the clinically significant efflux pumps belong to the MFS family. Examples include NorA in *Staphylococcus aureus* and PmrA in *Streptococcus pneumoniae* (Piddock et al. 1999). The ATP-binding cassette (ABC) transporter superfamily, another significant group, plays diverse roles in nutrient uptake, drug resistance, and cell signalling. The ABC transporter superfamily can be further divided into two main categories: uptake and efflux transport systems (Siarheyeva et al. 2007). One of the most well-studied ABC efflux transporters is the LmrA pump in *Lactococcus lactis*. This protein is responsible for pumping out toxic compounds, including antibiotics and detergents, from the cell. LmrA consists of six transmembrane domains (TMDs) and two nucleotide-binding domains (NBDs), making it a member of the ABC-A subfamily (Kumar et al. 2005). The *msr* (for macrolide streptogramin resistance) genes in *Staphylococci* enable an active efflux mechanism that causes resistance to macrolides, streptogramins, and azalides (Clancy et al. 1997; Leclercq et al. 2002). By clarifying the structure, function, and substrate specificity of these efflux systems, we can develop novel inhibitors or combination therapies to overcome multidrug resistance mediated by efflux.

### Deactivation of antibiotics

Enzyme-catalysed antibiotic inactivation is a significant factor in antibiotic resistance. Various resistant enzymes capable of degrading or modifying different types of antibiotics, including β-lactams, aminoglycosides, phenols, and macrolides, have been identified (Liu et al. 2021). The inactivation of β-lactam antibiotics by β-lactamases serves as a typical example of enzyme inactivation (Li et al. 2024). β-Lactamase is the primary mechanism of β-lactamase resistance in *Enterobacteriaceae* bacteria such as *P. aeruginosa* and *Acinetobacter baumannii*. Functionally, there are four classes of beta-lactamases (A-D); classes A, C, and D are serine β-lactamases, whereas class B members are zinc-dependent metallo-β-lactamases (Darby et al. 2023). Historically, the first β-lactamase, penicillinase, was identified in 1940, marking the beginning of efforts to understand this resistance mechanism. Since then, researchers have identified numerous β-lactamases capable of neutralizing the antibacterial activity of various β-lactam antibiotics. For instance, extended-spectrum β-lactamases (ESBLs) have evolved to inactivate advanced generations of β-lactam antibiotics, including cephalosporins (Chevalier et al. 2017). These enzymes have become highly prevalent, with the TEM family (discovered in 1965) now found in up to 60% of clinical isolates of *Enterobacteriaceae* and 50% of *Haemophilus influenzae* (Nazarov et al. 2022). Another notable β-lactamase, SHV-1, was first identified in 1972 and remains widespread in *E. coli* and *K. pneumoniae* isolates (Tsutsumi et al. 2019). The continual evolution of β-lactamase families underscores the dynamic nature of antibiotic resistance, highlighting the necessity of ongoing surveillance and characterization.

Currently, carbapenem resistance is a major concern, since carbapenems are highly effective antibiotics. The combination of carbapenem resistance with other β-lactam resistance can lead to the development of superbugs that are resistant to multiple classes of antibiotics (Lima et al. 2020). In 2017, the WHO listed three "critical" priority pathogens, all of which are resistant to carbapenems. Carbapenemases such as KPC (class A), NDM (class B), and OXA (class D), have the ability to hydrolyse penicillins, cephalosporins, and carbapenems, and significantly reduce the number of available drug treatment options for specific infections (Babu et al. 2020). The development of new antibiotics to replace those that are no longer effective is slow and costly. Thus, the development of new strategies to combat the rising tide of the carbapenem resistance is needed. In conclusion, enzyme-catalysed antibiotic inactivation is a major that contributed factor to antibiotic resistance. The identification and characterization of resistant enzymes can help inform the development of new strategies to combat antibiotic resistance.

### Modification of the target site

Some bacteria have developed the capacity to modify the structural composition of their antibiotic targets. This adaptation significantly diminishes the efficacy of antibiotics, since they can no longer effectively adhere to their intended targets in bacterial cells. Bacteria can reshape and chemically alter crucial molecules such as ribosomes, which are essential for protein synthesis. Consequently, antibiotics are rendered ineffective, which allows bacteria to proliferate unchecked. One notable example of this phenomenon is methicillin-resistant *Staphylococcus aureus* (MRSA), which is a frequent cause of hospital-acquired infections. MRSA produces an enzyme known as penicillin binding protein 2a (PBP2a), which modifies the structure of the target molecule intended for antibiotic binding (Sharma et al. 2023). This modification prevents the antibiotic from binding to the target and renders it ineffective against the bacterium. Similarly, *Mycobacterium tuberculosis*, the causative agent of tuberculosis, employs structural modifications of its target molecule, DNA gyrase, to resist the action of fluoroquinolone antibiotics. By altering the DNA gyrase structure, *M. tuberculosis* effectively prevents fluoroquinolones from interfering with its DNA replication process, allowing the bacterium to maintain pathogenicity despite antibiotic treatment (Shortridge et al. 1999). These examples illustrate a common strategy that bacteria use to evade antibiotics: structural alterations to essential molecules that serve as treatment targets.

Another important mechanism of antibiotic resistance involves modifications to bacterial ribosomes, which is particularly relevant for macrolide-lincosamide-streptogramin B (MLSB) antibiotics. In *Staphylococcus* species, the erm genes (*ermA* and *ermC*) encode enzymes that methylate the 23S rRNA subunit of the ribosome, thereby preventing the binding of MLSB antibiotics (Tait et al. 2000). This methylation alters the ribosomal structure, rendering the antibiotics ineffective. Additionally, the *cfr* gene, often located on conjugative plasmids, facilitates the spread of this resistance mechanism between bacterial species, exacerbating the global issue of antibiotic resistance (Berglund et al. 2017). Collectively, the ability of bacteria to modify their antibiotic targets represents a critical challenge in the treatment of bacterial infections. By understanding the molecular basis of these modifications, researchers can design more effective therapies that circumvent these resistance mechanisms and continue to provide effective treatments for bacterial infections.

## Combinations of TCM and antibiotics

Excessive antibiotic usage poses a potential threat of depleting effective antibiotic reserves. To counter the increasing antibiotic resistance and increase the efficacy of current treatments, a comprehensive drug strategy must be implemented. Evaluating combination therapies with precision is essential to discern synergistic or antagonistic effects and anticipate potential toxicological implications (Odds et al. 2003). Depending on the degree of synergy, effects can be categorized as additive or synergistic. Additive effects signify that the combined impact of drugs is equal to the sum of their individual effects. Synergistic effects indicate that the combined impact exceeds the sum of individual effects. Antagonism occurs when the combined drugs exhibit opposing effects, which reduces or make the efficacy negligible. The nature of the interaction varies with drug ratio and concentration and can be assessed through the Fractional Inhibition Concentration Index (FICI) using checkerboard analyses in microbiology laboratories (Gómara et al. 2019). The principle of Fractional Inhibitory Concentration (FIC) determination relies on the Minimum Inhibitory Concentration (MIC) and is the least concentration at which a compound on its own can inhibit microbial growth. The MIC of Compound A in the presence of B divided by the MIC of A alone yields the Fractional Inhibitory Concentration of A (FIC A), and the MIC of Compound B in the presence of A divided by the MIC of B alone yields FIC B. The FICI (fractional inhibitory concentration index) is the sum of FIC A and FIC B:

$$\text{FICI}=\frac{\text{MIC of drug A in combination}}{\text{MIC of drug A alone}}+\frac{\text{MIC of drug B in combination}}{\text{MIC of drug B alone}}$$

The combined effects are divided into four categories: synergistic effects (FICIs ≤ 0.5), antagonistic effects (FICIs > 4.0) and no interaction effects (FICIs > 0.5–4.0) (Odds et al. 2003).

Combination therapy often uses the synergy of multiple antimicrobial agents to improve the clinical results, particularly when multiple antibiotics are involved. However, there is little evidence that they can halt further development of antibiotic resistance. Some studies even indicate that adding synergistic antimicrobials at sub-inhibitory concentrations can accelerate the development of bacterial resistance (Chait et al. 2007; Hegreness et al. 2008). Consequently, continuous treatment of bacterial infection with synergistic combinations increases the number of ineffective antibiotics. Synergists can block or bypass the original resistance mechanisms, and reintroducing antibiotics through combination therapy can be a strategy to halt the development of bacterial resistance. Designing combination therapies requires a fundamental understanding of the antibiotic action spectrum and bacterial resistance mechanisms to enhance or broaden the antimicrobial activity against target species. The primary goals of antimicrobial treatment are to eradicate bacteria or inhibit their growth to enable the immune system to clear the infection. Depending on their mechanism of action, antibiotics can work against Gram-positive and/or Gram-negative bacteria or, occasionally, against both. The existing synergistic or additive drug combinations typically fall into three categories (Baym et al. 2016). The first is a combination of antibiotics, the second combines an antibiotic with a non-antibiotic substance with no direct antibacterial activity, and the third combines TCM with antibiotics, which is the focus of this review.

## Therapeutic effects of TCM/antibiotic combinations

**Fig. 2:**
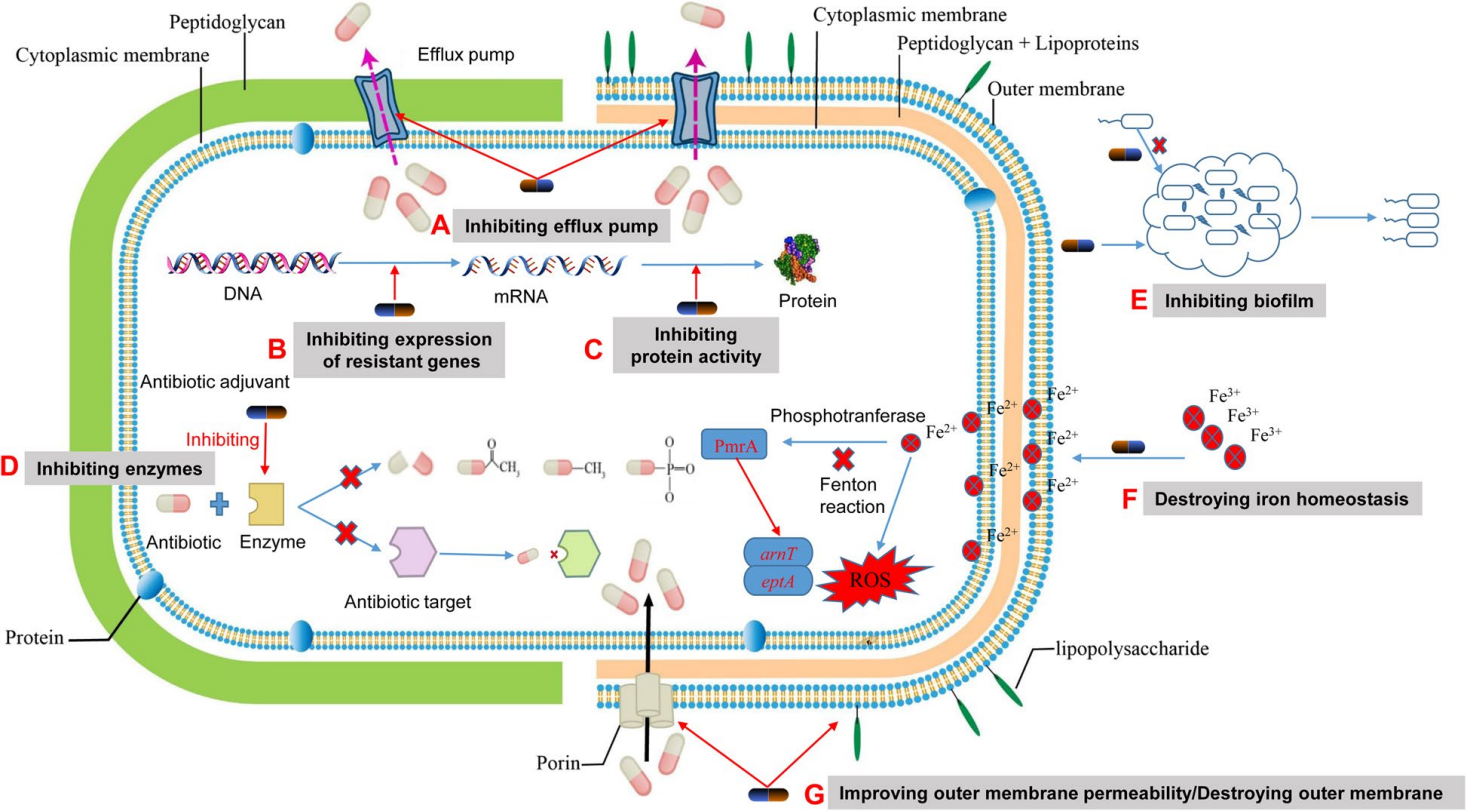
The mode of action of antibiotic adjuvants to enhance the efficacy of antibiotics. **A** Enhancing the intracellular antibiotic accumulation through inhibiting efflux pump activity; (**B**) Inhibiting expression of resistant genes; (**C**) Inhibiting protein activity; (**D**) Inhibiting of hydrolase/modifying enzyme on antibiotics or antibiotic targets; (**E**) Inhibiting the formation of biofilm or destroying biofilm; (**F**) Modulating the membrane charge of bacteria via disrupting iron homeostasis; (**G**) Enhancing the intracellular antibiotic accumulation through improving outer membrane permeability or destroying outer membrane

Increasingly many TCM/antibiotic combinations are globally investigated to advance the treatment of MDR bacterial infections. The antibiotics involved span various classes, including β-lactams (e.g., ampicillin), aminoglycosides (e.g., streptomycin), macrolides (e.g., erythromycin), lincomycin (e.g., lincomycin), and polypeptides (e.g., polymyxin) (Joung et al. 2016; Liu et al. 2000; Siriwong et al. 2015; Xu et al. 2017). These studies explore antibiotics that target both external and internal bacterial structures (Fig. 2). Antibiotics that target external structures such as bacterial cell membranes and cell walls may share common action sites with TCMs. In addition, TCMs exhibit antibacterial properties by inhibiting the beta-lactamase and metabolic activity-associated enzymes (Cai et al. 2018; Zuo et al. 2012). To better understand the variations in the combined efficacy of bacterial targets of antibiotics, all collected drug combinations were categorized into four groups: TCM/antibiotic combinations that target the cell membrane, TCM/antibiotic combinations that inhibit bacterial cell wall synthesis, TCM/antibiotic combinations that inhibit bacterial efflux pumps, and TCM/antibiotic combinations with new coordination mechanisms (Table 1).

**Table 1:** The synergistic mechanism of TCM monomer with antibiotics

Classification	TCM	Antibiotic	Strain	Resistance gene	FICI	Synergistic mechanism	Animal model	Ref
Alkaloid	Berberine	Gentamicin	*P. aeruginosa*	-	< 0.5	Damage the biofilm	Lung infection	(Li et al. 2017)
Alkaloid	Berberine	Ciprofloxacin	*Salmonella, K. pneumoniae*	-	0.375–1	Inhibit the formation of bacterial biofilm	-	(Shi et al. 2018)
Alkaloid	Berberine and chloride	Fusidic acid	MRSA	*fusA*	0.19–0.5	Damage formation of biofilm	-	(Liang et al. 2014)
Alkaloid	Total alkaloid	Cefotaxime, Ceftazidime	*E. coli*	*mexA*	≤ 0.5	Damage the biofilm	-	(Jaktaji et al. 2018)
Alkaloid	Berberine	Azithromycin	*P. aeruginosa*	-	0.13–0.5	Inhibition of bacterial MexXY efflux pump gene	-	(Li et al. 2017)
Alkaloid	Berberine	Imipenem	*P. aeruginosa*	-	0.375	Inhibition of bacterial MexXY-OprM efflux pump gene	-	(Su et al. 2018)
Alkaloid	Berberine hydrochloride	Tigecycline, sulbactam, meropenem and ciprofloxacin	*A. baumannii*	*adeB*	< 0.5	Reduced the efflux of antibiotics	Murine thigh infection model	(Li et al. 2021)
Alkaloid	Berberine hydrochloride	Fluconazole	*C. albicans*	-	0.03–0.06	Down-regulated efflux pump gene CDR1	-	(Yong et al. 2020)
Alkaloid	Piperine	Gentamicin	MRSA	-	0.5	Inhibition of bacterial efflux pump gene	-	(Khameneh et al. 2015)
Alkaloid	Baicalein	β -lactam antibiotics	*S. aureus*	-	≤ 0.5	-	-	(Liu et al. 2000)
Alkaloid	Berberine	Fluconazole	*C. albicans, C. tropicalis*	-	0.03–0.5	Inhibit the synthesis of ergosterol	-	(Xu et al. 2017)
Alkaloid	Berberine	Oxacillin	MRSA	-	0.5	Inhibit the MRSA adhesion to and intracellular invasion into HGFs	-	(Yu et al. 2005)
Alkaloid	Berberine	Azithromycin, Levofloxacin	MRSA	SCCmecIII	0.188–0.5	-	-	(Zuo et al. 2012)
Flavone	Tea-Derived Catechin Gallates	β-lactam	MRSA	-	-	Inhibit formation of biofilm	-	(Taylor et al. 2020)
Flavone	Kaempferol	Colistin	Gram-negative pathogens	-	0.012 to 0.375	Reduced the density of cellular arrangement of the biofilm	Survival of *G. mellonella*	(Zhou et al. 2022)
Flavone	Baicalein	Meropenem	*A. baumannii*	OXA-51, OXA-23	< 0.375–0.5	Inhibit formation of biofilm, increase permeability	-	(Guran et al. 2023)
Flavone	Baicalein	Linezolid	*MRSA*	-	-	Damage the biofilm and increase permeability	Biofilm infection	(Liu et al. 2020)
Flavone	Apigenin	Ceftazidime	*E. cloacae*	*ampC*	< 0.27	Damage the biofilm and cause leakage of intracellular components	-	(Eumkeb et al. 2013)
Flavone	Quercetin	Tetracycline	*E. coli*	-	< 0.5	Damage the biofilm and increase permeability	-	(Qu et al. 2019)
Flavone	Quercetin	Tobramycin, Amikacin	*P. aeruginosa*	-	0.25–0.5	Effectively penetrating the biofilm matrix, causing bacterial death	-	(Vipin et al. 2020)
Flavone	α-Mangostin	Ceftazidime	*A. Baumannii*	-	< 0.35	Membrane damage	-	(Pimchan et al. 2017)
Flavone	Reserpine, pyrrolidine, quinine, morin and quercetin	Ciprofloxacin, tetracycline and erythromycin	*S. aureus*	*NorA*	0.03125- 0.5	Biofilm prevention and/or control, Inhibit NorA efflux pumps on the biofilm	-	(Abreu et al. 2016)
Flavone	Baicalein (5, 6, 7-trihydroxyflavone)	Tetracycline	MRSA	*tetK*	0.03125–0.125	Inhibition of bacterial tetK efflux pump gene	-	(Fujita et al. 2005)
Flavone	Baicalein	Ciprofloxacin	MRSA	*NorA*	0.02–0.5	Inhibits the production of NorA efflux pump gene and pyruvate kinase	-	(Chan et al. 2011)
Flavone	Baicalein	Oxytetracycline, Tetracycline,	MRSA	-	≤ 0.5	Inhibition of bacterial tetK efflux pump gene	-	(Novy et al. 2011)
Flavone	Daphne genkwa Siebold	Gentamicin	MRSA	-	-	PBP2a and PBP4 inhibition	-	(Fan et al. 2023)
Flavone	Baicalein	Penicillins	MRSA	*blaZ* and *mecA*	0.14- 0.38	Decreased bacterial activity of penicillinase	-	(Qian et al. 2015)
Flavone	α-Mangostin	Oxacillin	*S. saprophytic*	-	0.37	-	-	(Phitaktim et al. 2016)
Flavone	Galangin and kaempferide	Amoxicillin	*E. coli*	-	≤ 0.5	Inhibition of β-lactamase activity and peptidoglycan structure	-	(Eumkeb et al. 2012)
Flavone	Morin	Oxacillin	MRSA	-	0.06–0.5	Inhibition of cell-wall synthesis	-	(Mun et al. 2015)
Flavone	Quercetin	Meropenem	*P. aeruginosa, A. baumannii, E. coli,*	-	0.093–0.5	Decreased expression of acrB and disrupted the bacterial cell wall	-	(Pal et al. 2020)
Flavone	Luteolin	Ampicillin, Oxacillin,	MRSA	-	0.125–0.562	-	-	(Joung et al. 2016)
Flavone	Luteolin, Quercetin	Ceftazidime	*S. pyogenes*	-	0.37, 0.27	Inhibited peptidoglycan synthesis and increased biofilm permeability	-	(Siriwong et al. 2015)
Flavone	Silibinin	Kanamycin	*S. aureus*	-	-	Reduce H_2_O_2_ levels	Mouse peritonitis	(Cai et al. 2018)
Flavone	Myricetin, 7,8-dihydroxyflavone, and luteolin	Colistin	Gram-negative pathogens	*pmrA*/*pmrB*	0.125–0.458	Disrupt bacterial iron homeostasis	Mouse salmonellosis	(Zhong et al. 2023)
Phenylpropanoids	Cinnamic and pcoumaric acid	Amikacin, ampicillin, ciprofloxacin, and vancomycin	*E. coli, E. aerogenes*, *P. aeruginosa* and *S. aureus*	-	0.14–0.5	Damage the biofilm	-	(Hemaiswarya et al. 2010)
Phenols	Pterostilbene	Gentamicin	*S. aureus*, *E. coli*	-	0.125–0.25	Damage to the structural integrity of the cells	-	(Lee et al. 2017b)
Phenols	Epigallocatechin gallate	Tetracycline	*S. aureus*	Tet(K)	0.0625–0.5	Inhibition of bacterial tetK and tetB efflux pump gene	-	(Roccaro et al. 2004)
Phenols	Epigallocatechin gallate	Penicillin, Ampicillin	*S. aureus*	-	≤ 0.5	Inhibit the action of penicillinase	-	(Zhao et al. 2002)
Phenols	Proanthocyanidins (cPAC)	Oxacillin and carbenicillin	MRSA	*blaZ*	0.125–0.5	Hindering the synthesis bacterial cell wall	-	(Gallique et al. 2021)

### TCM/antibiotic combinations targeting the cell membrane

The effectiveness of antibiotics that target intracellular components depends heavily on the ability of the antibiotic to penetrate the bacterial cell membrane. Hydrophobic antibiotics can diffuse through lipid bilayers, but hydrophilic antibiotics require specific bacterial pore proteins for entry. TCM has shown promise in enhancing the membrane permeability and drug uptake, thereby improving the efficacy of antibiotics against drug-resistant bacteria. Certain compounds, including phenols, flavonoids, and quinones, have been shown to increase cell membrane permeability, which raises antibiotic concentrations within bacteria and ultimately leads to bacterial death (Li et al. 2022). This increased permeability has been demonstrated in vitro in both Gram-positive bacteria (such as *Enterococcus* and *S. aureus*) and Gram-negative bacteria (such as *A. baumannii*, *E. coli*, and *P. aeruginosa*) (Lee et al. 2017a, b; Liu et al. 2020; Zhou et al. 2022). For example, the flavonoid compound kaempferol enhances the membrane permeability by reducing the cell arrangement density in Gram-negative bacteria, and thereby increasing the antibacterial activity of colistin (Zhou et al. 2022). The structural analysis revealed that the cells treated with α-mangostin/ceftazidime had an increased volume, increased permeability in their cytoplasm and outer membrane, and reduced outer membrane peptidoglycan-associated protein bands (Pimchan et al. 2017).

In addition to their ability to increase membrane permeability, some compounds also exhibit direct antibacterial activity by damaging the bacterial membrane. Several compounds such as polyphenols and alkaloids have shown direct antibacterial activity against bacteria by causing membrane damage (Pourahmad et al. 2018; Shi et al. 2018). The alkaloid berberine and ciprofloxacin have a synergistic effect against *Salmonella*. Further investigations revealed that the downregulation of *luxS*, *rpoE*, and *ompR* was mainly associated with the formation of hair, cellulose, crimp, flagellin, and virulence factors, affected the membrane thickness and monolayer size, inhibited the biofilm formation in a dose-dependent manner, and even destroyed biofilms (Shi et al. 2018). Furthermore, the combined application of sandalwood and gentamicin resulted in similar activity against *E. coli* and *S. aureus* and formed a rough membrane surface with visible pores and bubbles (Lee et al. 2017a, b). These findings indicate that herbal plant extracts may enhance the antibacterial effects of traditional antibiotics, providing a novel avenue for combating drug-resistant infections.

### TCM/antibiotic combination inhibits the bacterial cell wall synthesis

Β-lactam and glycopeptide antibiotics are known to deactivate bacteria by blocking cell wall synthases, specifically penicillin-binding proteins and transpeptidases. Various studies have explored the use of TCM monomers in combination with β-lactams or glycopeptides to increase the deactivation of bacterial pathogens. These pathogens include gram-positive bacteria such as *S. aureus* and Gram-negative bacteria such as *P. aeruginosa*, *A. baumannii*, *E. coli*, and *K. pneumoniae* (Eumkeb et al. 2013; Fan et al. 2023; Gallique et al. 2021; Pal et al. 2020). For example, studies have shown that α-mangosteins derived from mangosteen peel can enhance the antibacterial effect of oxacillin against *Staphylococcus*. The possible reason is the formation of a complex between the isolated α-mangosteins and β-lactamase type IV, which deactivates the β-lactamase activity (Eumkeb et al. 2013). This interaction is similar to the effects of homogannin and epigallocatechin gallate (EGCG), which inhibit β-lactamase and penicillinase activity by interacting with α-gostein-like pyranoid rings (Denny et al. 2002; Zhao et al. 2002).

Other studies have reported that EGCG enhances antibacterial activities by inhibiting the penicillinase activity (Zhao et al. 2002) and disrupting the membrane structure (Palacios et al. 2014). Furthermore, EGCG can reduce the fluidity of the bacterial membrane (Bernal et al. 2010), which can affect the activity of transpeptidase. These findings suggest that EGCGG may have potential as an adjuvant therapy in combination with β-lactam antibiotics to treat *S. aureus* infections. Quinazolinones enhance piperacillin-tazobactam by inducing conformational changes in PBP2a (Janardhanan et al. 2019). The bactericidal activity of the quercetin-Meropenem combination may be due to alterations in the expression of *bla*_VIM_ (carbapenase) and *ompC*, which undermines the integrity of the cell wall (Fan et al. 2023). Overall, these studies highlight the potential of combining TCM compounds with antibiotics to inhibit bacterial cell wall synthesis and enhance antimicrobial efficacy, offering a promising strategy for combating MDR bacterial infections.

### TCM/antibiotic combination inhibits the efflux pump activity

The efflux pump, which is a protein transporter on the plasma membrane of the cell, is integral to maintaining the internal environment of bacteria. Its functions include assisting bacteria in expelling toxins and antibiotics, which is a critical feature that contributes to drug resistance in numerous bacteria. Bacterial efflux transporters are categorized into five families: RND, ABC, MATE, MFS, and SMR; their presence is prevalent in both Gram-positive and Gram-negative bacteria (Blanco et al. 2016).

Current research indicates that RND pumps are largely responsible for the resistance to chloramphenicol, beta-lactam, fluoroquinolone, and fusidic acid. The overexpression of efflux pumps, particularly Mex-oprM, confers antibiotic resistance to *P. aeruginosa* (Glavier et al. 2020). One strategy to restore antibiotic activity involves combining TCM monomers with existing antibiotics (Schmidt et al. 2016). Clinical isolates of *P. aerugino*sa harbouring MexAB-OprM, MexD-OprJ, and MexEF-OprN efflux pump mutations were tested using a combination of conessine and levofloxacin (Horna et al. 2018). The efficacy of levofloxacin alone and in combination with alkaloids was assessed through susceptibility tests and time-kill studies. The results demonstrated that conessine enhanced the efficacy of levofloxacin against the efflux-pump-mediated resistance in *P. aeruginosa* (Siriyong et al. 2018). Similarly, conessine exhibited a synergistic effect when it was combined with novobiocin or rifampicin to treat extensively resistant *A. baumannii* infections (Siriyong et al. 2016). Anti-malarial compounds such as quinoline alkaloids, dictamnine, and masculine compounds are known for their significant antibacterial activity (Kingston et al. 2022). The combination of the ergot alkaloid chanoclavine with tetracycline can substantially inhibit the growth of resistant *E. coli* (Dwivedi et al. 2019). Although chanoclavine alone shows no antibacterial activity, it displays significant synergy with tetracycline. This effect is attributed to the inhibition of the ATPase-dependent bacterial efflux pump by chanoclavine.

Baicalein can notably reverse the resistance of MRSA to ciprofloxacin by inhibiting the NorA efflux pump in vitro (Abreu et al. 2016). Baicalein can inhibit the activity of MRSA pyruvate kinase and potentially decrease the ATP levels, which further enhances the antibacterial effect of baicalein on MRSA (Chan et al. 2011). Berberine can decrease the resistance to aminoglycosides in *P. aeruginosa* containing the MexXY efflux system, which indicates that it is a potential novel inhibitor of MexXY-dependent aminoglycoside efflux in *P. aeruginosa* (Morita et al. 2016). Tetracycline efflux, which is responsible for high levels of tetracycline resistance, is energy-dependent and functions through two distinct types of efflux pumps: multi-drug-resistant pumps and tetracycline-specific transporters from the *tet* gene family (Fujita et al. 2005; Berglund et al. 2020). Studies have shown that EGCG and quercetin can significantly sensitize *Staphylococcus* to the effects of tetracycline and effectively reverse resistance in strains that have developed resistance due to *Tet(K)* or *Tet(B)* efflux pump protein expression (Novy et al. 2011; Qu et al. 2019). Therefore, the search for efflux pump inhibitors is a potential strategy to overcome bacterial resistance (Li et al. 2021; Yong et al. 2020).

### New coordination mechanism

Colistin is an antibiotic to eliminate multi-drug-resistant bacteria, notably carbapenem-resistant Enterobacter (Biswas et al. 2012). Despite its rapid bacteria-clearing effect, approximately 70% of patients do not respond well to colistin treatment (Falagas et al. 2010; Sabnis et al. 2021). Its nephrotoxicity also prevents its excessive use (Liu et al. 2016; Satlin et al. 2020). The emergence of colistin resistance, such as MCR-1, exacerbates this situation and necessitates the urgent development of colistin adjuvants (Liu et al. 2016). However, researchers have reported that the prenylation of the flavonoid phenol skeleton is essential for its antibacterial activity (Song et al. 2021). Previous studies revealed that catechol modifications enabled 7,8-dihydroxyflavone, myricetin, and luteolin to regulate iron and related responses in bacteria (Zhong et al. 2023). These flavonoids disrupt the bacterial iron homeostasis, which enhances the colistin binding and ROS production by dysregulating iron signalling. Flavonoid-induced iron deficiency prevents *pmrA* from being activated through phosphorylation, which inhibits *arnT* and *eptA* at the transcriptional level. As a result, colistin can more efficiently target lipids in the absence of positively charged molecules such as pEtN. Increased colistin binding disrupts the bacterial membrane and promotes the production of lethal ROS by interfering with the electron transport chain (Choi et al. 2017; Deris et al. 2014). Overall, these findings highlight flavonoid-induced iron deficiency as a promising strategy to enhance colistin's effectiveness and combat MDR bacterial infections.

## Conclusions and future perspectives

The excessive use of antibiotics to combat bacterial infections has exacerbated antibiotic resistance and necessitates urgent innovations in treatment methods. Considering the formidable expenses and high failure rates associated with developing new antibiotics, combination therapy has emerged as a compelling alternative. This approach holds potential for devising novel treatment regimens in the post-antibiotic era. Unlike monotherapy, combination therapies offer extensive pathogen coverage, particularly against unidentified or novel pathogens. Moreover, significant strides in high-throughput molecular screening techniques, including proteomics, metabolomics, genomics, and network pharmacology, have greatly enhanced the understanding and characterization of numerous antimicrobial phytochemicals. Many mechanisms of action have been elucidated to pave the way for synergistic combinations with antibiotics as a promising strategy against MDR pathogens.

Among the array of promising traditional Chinese medicines (TCMs) under study, polyphenols, flavonoids, and alkaloids have emerged as notable candidates individually or in combination. They exhibit synergistic potential when paired with conventional antibiotics, where beta-lactam antibiotics are the most extensively researched. The primary mechanisms of synergy involve inhibiting efflux pumps and β-lactamase and enhancing the membrane penetration. The strategic screening of suitable TCMs holds promise for significantly decreasing the drug development timelines, particularly in terms of clinical safety evaluations. However, integrating TCMs with antibiotics presents challenges, especially with respect to the precise determination of combined dosages. Additionally, identifying specific targets that are responsible for the antimicrobial activity of TCMs remains a pressing issue (Drusano et al. 2015; Gómara et al. 2019). The criticality of determining the correct combined dosage cannot be overstated, since the efficacy of drug interactions is intricately tied to dosing. Incorrect antibiotic combinations can compromise the effectiveness and potentially foster resistance. For example, mismatched pharmacokinetic properties can restrict the drug exposure to target tissues, which hinders the translation of in vitro findings to clinical efficacy. Thus, establishing optimal dosing regimens is pivotal for clinical application. Although combination therapy can enhance the therapeutic outcomes, it also necessitates rigorous toxicological assessments prior to clinical trials to mitigate potential toxicity risks (Benesic et al. 2019). Overall, combination therapy, particularly antibiotic-TCM therapy, is a promising frontier for combating multi-drug-resistant infectious diseases.

## Data Availability

Not applicable.
